# Pooled prevalence of anxiety and depressive symptoms among adults with type 2 diabetes: a systematic review and meta-analysis

**DOI:** 10.1007/s40200-026-02079-z

**Published:** 2026-09-24

**Authors:** Rosiane R. Silva, Maurício S. da Costa, Luana A. L. Almeida, Poliany P. Cruz, Robervânia R. Silva, Alessandro C. Alves, Ricardo A. L. de Sousa, Ricardo C. Cassilhas

**Affiliations:** 1 Department of Physical Education, School of Biological and Health Sciences (FCBS), Federal University of the Jequitinhonha and Mucuri Valleys (UFVJM), Diamantina, MG Brazil; 2 Neuroscience and Exercise Study Group (GENE), UFVJM, Diamantina, MG Brazil; 3 Graduate Program in Health Sciences (PPGCS), UFVJM, Diamantina, MG Brazil; 4 Department of Behavioral Neuroscience, Oregon Health & Science University, Portland, OR USA; 5 Graduate Program in Health Sciences (PPGCS), Montes Claros State University, Montes Claros, MG Brazil; 6 Federal University of the Jequitinhonha and Mucuri Valleys (UFVJM), Diamantina, MG Brazil; 7 Department of Health, Physical Education, and Recreation, Chicago State University, Chicago, USA

**Keywords:** Type 2 diabetes mellitus, Anxiety, Depression, Depressive symptoms, Mental health

## Abstract

**Purpose:**

Anxiety and depressive symptoms are highly prevalent among individuals with type 2 diabetes mellitus (T2DM), yet they remain underdiagnosed despite their substantial impact on health outcomes. In addition to impairing treatment adherence, these conditions may contribute to the development of chronic complications, reduce quality of life, and, in severe cases, increase mortality. Therefore, this review aims to estimate the pooled prevalence of anxiety symptoms and depressive symptoms among adults with T2DM and to explore potential sources of between-study heterogeneity, including assessment instruments and cut-off points.

**Methods:**

PubMed, Embase, PsycINFO, Scopus, and Web of Science were searched from inception to July 6, 2026. After screening 6,948 records according to PRISMA 2020, 34 cross-sectional studies involving 21,024 participants were included. We considered observational studies involving adults with T2DM that reported the prevalence of anxiety symptoms or depressive symptoms assessed using validated screening instruments or diagnostic criteria. Two reviewers independently screened the records, extracted the data, and assessed the risk of bias. Separate random-effects meta-analyses were conducted for anxiety symptoms and depressive symptoms.

**Results:**

Thirty-four cross-sectional studies involving 21,024 participants were included. The pooled prevalence was 43.82% for anxiety symptoms (95% CI 36.86–50.90; $\:{I}^{2}=99.06\%$) and 41.83% for depressive symptoms (95% CI 33.83–50.06; $\:{I}^{2}=99.31\%$). Estimates varied substantially across assessment instruments and cut-off points. Several studies reported associations with sex, glycemic control, and social support; however, differences in the definitions, measurements, and analytical methods prevented quantitative pooling of these factors.

**Conclusion:**

Anxiety and depressive symptoms were frequently reported among adults with T2DM. However, the extreme between-study heterogeneity limits the interpretation of the pooled estimates, which should be understood as summary measures across diverse study populations rather than universal prevalence estimates. These findings should not be interpreted as estimates of clinically diagnosed anxiety or depressive disorders.

**Supplementary Information:**

The online version contains supplementary material available at https://doi.org/10.1007/s40200-026-02079-z.

## Introduction

Type 2 diabetes mellitus (T2DM) is a chronic noncommunicable disease characterized by insulin resistance and/or impaired insulin secretion by pancreatic beta cells. It is associated with obesity and aging [1–3]. The global prevalence of diabetes has increased steadily over recent decades. In 2021, approximately 536.3 million people were living with diabetes worldwide. By 2024, the number had reached 584 million adults aged 20–79 years, with projections suggesting it will rise to 853 million by 2050 [2].

Chronic conditions such as T2DM are frequently associated with anxiety and depressive symptoms, as well as clinically significant depressive and anxiety disorders. However, the magnitude of this association varies across populations and measurement approaches [4–6]. A population-based cross-sectional study in Brazil showed that chronic disease was associated with higher odds of depressive symptoms and that multimorbidity further increased this risk compared with healthier individuals [4]. These findings support the integration of mental health screening into routine diabetes care to mitigate the adverse effects of these comorbidities on prognosis, quality of life, and metabolic control.

This comorbidity is particularly concerning because, in individuals with T2DM, anxiety and depressive symptoms may compromise treatment adherence, worsen self-management, impair glycemic control, and contribute to diabetes-related complications, including microvascular and macrovascular outcomes [7]. A study conducted with 2,955 outpatients with T2DM observed prevalence estimates of anxiety and depressive symptoms, along with a strong association between neuropathy and depressive symptoms (OR = 1.95; 95% CI 1.42–2.67; *p* < 0.001) [8].

Although the psychological burden associated with type 2 diabetes (T2D) has been examined in previous reviews, the available literature remains fragmented in its assessment of outcomes, populations studied, and measurement strategies. An umbrella review published in 2025, which synthesized 23 systematic reviews comprising 649 unique primary studies, demonstrated wide variation in the prevalence of depression among people with T2D and showed that these estimates differ according to geographic region and the method used to assess depression [9]. Regarding anxiety, a meta-analysis published in 2026 pooled 24 studies and reported a combined prevalence of 30%; however, the study population was restricted to individuals with T2D in India and exhibited high heterogeneity and differences in the assessment instruments used [10]. In this context, there remains a need for an international synthesis that estimates the prevalence of anxiety and depression in adults with T2D, using a consistent methodological framework and population, and systematically explores key sources of heterogeneity. The present review aims to address this gap by combining the two outcomes, applying a prevalence meta-analysis, analyzing potential associated predictive factors, and investigating the influence of assessment instruments and their respective cut-off points on the resulting estimates. To our knowledge, no previous review has quantified pooled prevalence estimates for both anxiety symptoms and depressive symptoms among adults with T2DM across multiple geographic regions using unified eligibility criteria and a single meta-analytic framework.

Despite the growing body of evidence on the relationship between T2DM and mental health conditions, important gaps remain, particularly regarding underdiagnosis and the limited incorporation of these outcomes into routine care. This gap warrants further synthesis of evidence to clarify the burden and associated factors of these comorbidities in T2DM. Therefore, this study aimed to summarize the reported prevalence of anxiety and depressive symptoms among individuals with T2DM and to examine associated factors. By quantifying pooled prevalence estimates for anxiety and depressive symptoms and by examining how these estimates vary across assessment instruments, cut-off points, and clinical settings, this review provides a more integrated picture of the psychological burden associated with T2DM than previous syntheses.

## Methodology

The review protocol was registered in PROSPERO (CRD42025635542), and the methods followed the Joanna Briggs Institute Reviewers Manual.

### Inclusion and exclusion criteria

We included observational studies reporting the prevalence of anxiety and/or depressive symptoms among adults aged 18 years or older of both sexes with T2DM. In this review, T2DM was defined as a heterogeneous group of metabolic disorders characterized by chronic hyperglycemia resulting from impaired insulin secretion, impaired insulin action, or both [1]. No restrictions were applied regarding language or publication period.

Animal studies and studies involving other types of diabetes were excluded. Interventional studies, systematic reviews, meta-analyses, case reports, and articles that did not use validated screening or diagnostic instruments or scales were excluded. Studies reporting prevalence data for only one of the two outcomes (anxiety or depressive symptoms) were also excluded, as both outcomes had to be assessed within the same sample to allow consistent comparisons across the pooled analyses.

### Search strategy

We searched PubMed, Embase, PsycINFO, Scopus, and Web of Science on July 6, 2026, using strategies tailored to each platform. The search strategy was structured around three conceptual blocks: population (type 2 diabetes mellitus), outcome (symptoms of anxiety and depression), and epidemiological context (prevalence, epidemiology, and cross-sectional studies), combined using the Boolean operator AND. In contrast, synonymous terms within each block were combined using OR. Controlled descriptors (MeSH/ Emtree, where available) and free-text terms related to the concepts of interest were employed. (see Supplementary Material 1). Searches were not restricted to Title/Abstract fields; controlled vocabulary terms and indexing fields were fully utilized whenever available. Outcome-related terms for anxiety and depressive symptoms were combined within the same search framework, and broader mental-health terminology was included to capture studies that focused predominantly on one of these outcomes. The complete database-specific search strategies, including all keywords, controlled vocabulary terms, search fields, and search dates, are provided in Supplementary Material 1.

### Study selection

All records were imported into Rayyan, and duplicates were removed. Titles and abstracts were independently screened by two reviewers, followed by full-text assessment. Disagreements were resolved through discussion or consultation with a third reviewer.

### Data extraction

Two independent reviewers extracted the data using a predefined form, and disagreements were resolved by consensus. The extracted information included authors, study type, context (location and recruitment source), number of participants, methods used to assess depression and anxiety symptoms, and combined prevalence and associated factors.

Regarding the reporting of associated factors, which varied considerably across studies, we pre-specified age, sex, BMI, and diabetes-related complications as core variables for structured synthesis. Other variables, such as disease duration, glycemic control, marital status, education, and income, were summarized descriptively when at least two studies reported them using comparable definitions.

### Risk of bias assessment

Two independent reviewers also assessed the risk of bias of each included study using the Joanna Briggs tool, a validated 9-item tool for assessing the methodological quality of prevalence studies [11]. A third reviewer was consulted in cases of disagreement. Methodological quality was classified as high (7–9 criteria met), moderate (5–6 criteria met), or low (< 5 criteria met).

### Statistical analysis

Statistical analyses were performed using MedCalc^®^ software version 23.7.1. The pooled prevalence of anxiety and depression in patients with T2DM was calculated using a random-effects model with a 95% confidence interval (CI) and presented in a forest plot. Statistical heterogeneity among studies was assessed using the I² statistic. Potential publication bias was investigated through visual inspection of funnel plots and Begg’s and Egger’s tests, with p-values < 0.05 considered indicative of statistically significant asymmetry. A sensitivity analysis was conducted by excluding studies one by one and by subgroups based on the instruments used and the cutoff point to classify anxiety and depression.

## Results

### Selection of studies

**Fig. 1:**
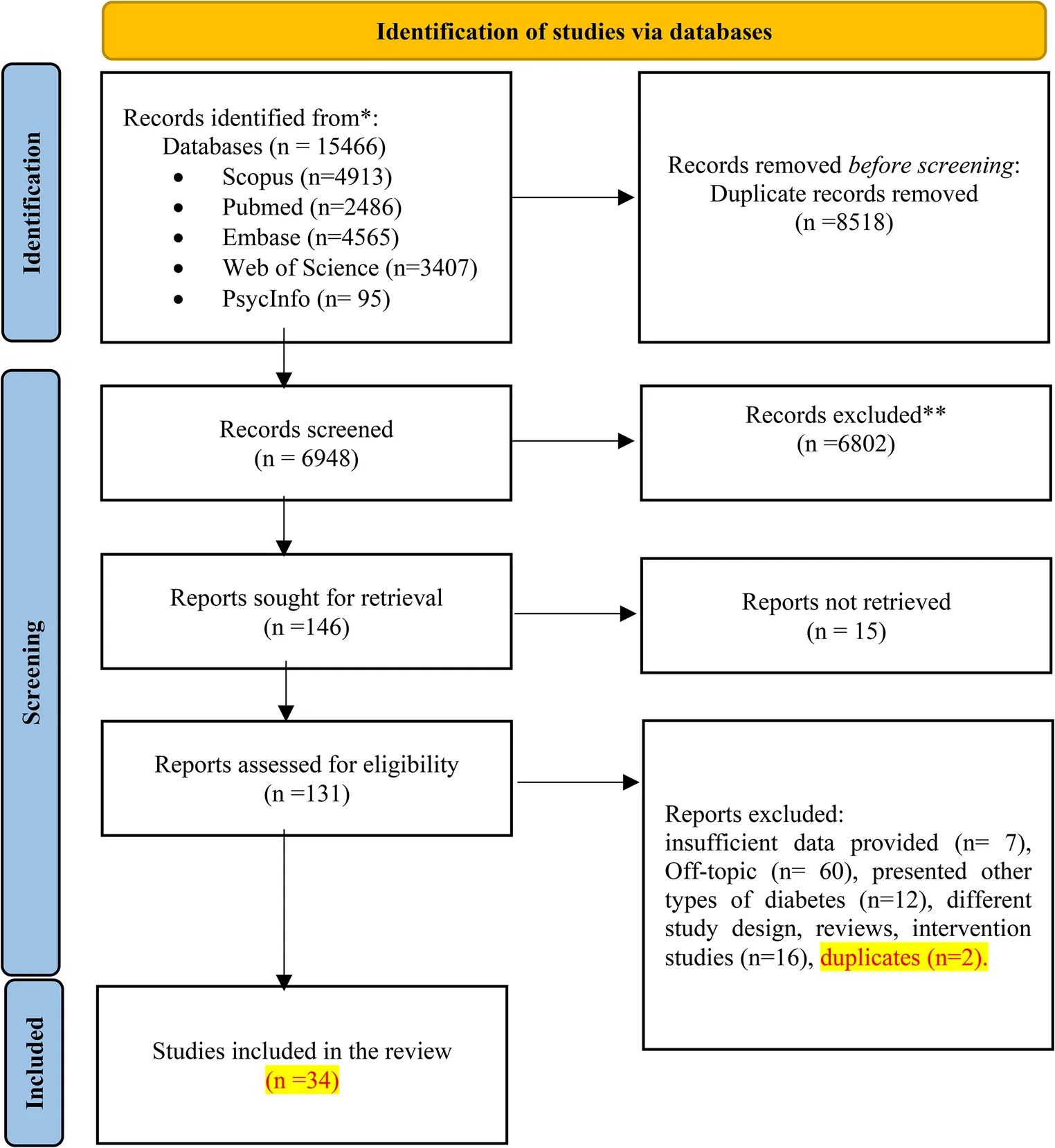
Flowchart for study selection according to PRISMA 2020 recommendations. n, number of records/reports at each stage

A total of 15,466 records were identified, of which 8518 duplicates were removed. After screening, 131 articles were assessed in full, and 34 studies were included. The reasons for excluding full-text articles are shown in Fig. 1.

### Characteristics of the included studies

Thirty-four studies published between 2010 and 2026 were included; all used a cross-sectional design and were conducted in Africa, the Americas, Europe, and Asia.

Characterization of the 34 included studies revealed an aggregate sample of 21,024 individuals with T2DM of both sexes, with sample sizes ranging from 62 to 2,730 participants. Regarding the demographic profile, the majority of studies (*n* = 18) included a higher proportion of women and were conducted in hospital settings (*n* = 15) (Supplementary Material 2). A wide disparity was observed in the prevalence rates of anxiety and depression, ranging from 16.4% to 84.9% and 3.3% to 88.4%, respectively, highlighting the diversity of the clinical contexts analyzed.

Different instruments were used to assess anxiety and depressive symptoms across the 34 included studies. For anxiety, the Hospital Anxiety and Depression Scale (HADS) was the most frequently used instrument, applied in 12 of the 34 studies (35.29%). For depression, the Patient Health Questionnaire (PHQ-9) was used in 12 of the 34 studies (35.29%) (Supplementary Material 2).

Table 1 presents the characteristics of the studies included in this review, as well as the prevalence of anxiety and depressive symptoms.

**Table 1:** Characteristics of the included studies (*n* = 34)

Studies	Country	Sample	Prevalence anxiety *n* (%)	Prevalence of depression *n* (%)
Albai et al. 2024 [12]	Romania	209	108 (51.7)	120 (57.5)
Aldebani et al. 2024 [13]	Saudi Arabia	412	194 (47)	224 (54.4)
Aljohani et al. 2022 [14]	Saudi Arabia	602	207 (34.4)	20 (3.3)
ALkhayyal et al. 2025 [15]	Saudi Arabia	391	332 (84.9)	346 (88.4)
Al-Mohaimeed, 2017 [16]	Saudi Arabia	300	131 (43.6% 95% CI: 37.9–49.3%)	104 (34.8% 95% CI: 29–40%)
Alzahrani et al. 2019 [17]	Saudi Arabia	450	171 (38)	152 (33.8)
Baxi et al. 2025 [18]	India	200	60 (30)	62 (31)
Butt et al. 2025 [19]	Pakistan	216	44 (20.4)	54 (25)
Cardenas et al. 2022 [20]	Ecuador	208	70 (33.7%, 95% CI 27.3–40.5)	66 (31.7%, 95% CI 25.4–38.5%)
Dehesh et al. 2020 [21]	Iran	1500	930 (62% 95% CI: 59.51–66.27)	885 (59% 95% CI: 54.48–63.12)
Elnaem et al.2025 [22]	Indonesia and Malaysia	606	252 (41.6)	343 (56.6)
Ganasegeran et al. 2014 [23]	Malaysia	169	53 (31.4)	68 (40.3)
Gashi et al. 2023 [24]	Kosovo	596	489 (82)	441 (74)
Kaur et al. 2013 [25]	Malaysia	2508	768 (30.5)	376 (15)
Khan et al. 2019 [26]	Pakistan	142	72 (50.7)	70 (49.2)
Khuwaja et al. 2010 [27]	Pakistan	889	514 (57.9 95% CI: 54.7–61.2)	387 (43.5 95% CI: 40.3–46.8)
Kintzoglanakis et al. 2022 [28]	Greece	182	32 (17.7)	30 (16.6)
Masmoudi et al. 2013 [29]	Tunisia	62	25 (40.3)	14 (22.6)
Mikaliukstiene et al. 2014 [30]	Lithuania	1022	433 (42.4)	291 (28.5)
Mohana et al. 2023 [31]	Somalia	100	30 (30)	23 (23)
Nigussie et al. 2023 [32]	Ethiopia	416	168 (40.4)	176 (42.3)
Pardhan et al. 2024 [33]	Bangladesh	600	132 (22)	186 (31)
Paudel et al. 2023 [34]	Nepal	283	89 (31.4)	103 (36.4)
Palizgir et al. 2013 [35]	Iran	184	128 (69.6)	130 (70.7)
Riise et al. 2025 [36]	Norway	1954	320 (16.4)	238 (12.2)
Rosas-Matias et al. 2019 [37]	Peru	327	107 (32.72)	78 (23.85)
Salih et al. 2022 [38]	Iraq	380	301 (79.2)	302 (79.5)
Sayin et al. 2019 [39]	Turkey	636	308 (48.4)	428 (67.3)
Sharma et al. 2021 [40]	Nepal	296	147 (49.7)	171 (57.8)
Subramanian et al. 2024 [41]	India	450	281(62.4)	328 (72.9)
Sun et al. 2016 [42]	China	893	501 (56.1)	389 (43.6)
Tamene et al. 2026 [43]	Ethiopia	409	148 (36.2)	208 (50.9)
Tovila- Zárate et al. 2012 [44]	Mexico	702	388 (55.10% 95% CI: 51.44–58.93)	335 (48.27% 95% CI : 44.48–52.06)
Tripathi et al. 2026 [45]	India	2730	472 (17.3)	554 (20.3)

*n* number of cases, *CI *confidence interval, *ND* not available. Values in parentheses represent percentages unless otherwise indicated as 95% CI

### Methodological quality

In Table 2, the mean methodological quality score was 7 out of 9 points (range, 5–9). Methodological concerns were identified (Table 2). Of the 34 studies included, 24 were classified as high quality, 10 as moderate quality. The main limitation was that ten studies (29.41%) were unclear for item 2 (Were study participants recruited appropriately? ). In 30 studies (88.24%), item 7, related to data collection reliability, was unclear; in 14 studies (41.18%) and 9 studies (26.47%), items 4 and 3, respectively, were unclear (Table 2).

**Table 2:** Methodological quality of the included studies (*n* = 34), assessed using the Joanna Briggs Institute critical appraisal tool for prevalence studies

Studies	Items									
	1	2	3	4	5	6	7	8	9	overall score (0–9)
Albai et al. 2024 [12]	y	y	y	y	y	y	nc	y	**y**	8
Aldebani et al. 2024 [13]	y	y	y	y	y	y	**y**	y	**y**	9
Aljohani et al. 2022 [14]	y	y	y	nc	y	nc	nc	y	nc	5
Alkhayyal et al. 2025 [15]	y	nc	nc	nc	nc	y	nc	y	y	5
Al-mohaimeed, 2017 [16]	y	y	y	nc	y	y	nc	y	y	7
Alzahrani et al., 2019 [17]	y	nc	y	y	y	y	nc	y	y	7
Baxi et al. 2025 [18]	y	y	y	y	y	y	nc	y	y	8
Butt et al. 2025 [19]	y	y	y	y	y	y	nc	y	y	8
Cardenas et al. 2022 [20]	y	y	y	nc	y	y	nc	y	y	7
Dehesh et al. 2020 [21]	y	y	y	nc	y	y	nc	y	y	7
Elnaem et al.2025 [22]	y	y	y	nc	y	y	nc	y	y	7
Ganasegeran et al. 2014 [23]	y	nc	nc	y	y	y	nc	y	y	6
Gashi et al. 2023 [24]	y	y	y	nc	y	y	nc	y	y	7
Kaur et al. 2013 [25]	y	y	y	nc	y	y	nc	y	y	7
Khan et al. [26]	y	nc	nc	y	nc	y	nc	y	y	5
Khuwaja et al. 2010 [27]	y	y	y	nc	y	y	nc	y	y	7
Kintzoglanakis et al. 2022 [28]	y	nc	nc	y	y	y	nc	y	y	6
Masmoudi et al. 2013 [29]	y	nc	nc	y	y	y	nc	y	y	6
Mikaliukstiene et al. (2014) [30]	y	nc	y	nc	nc	y	nc	y	y	5
Mohana et al. 2023 [31]	y	nc	nc	y	y	y	nc	y	y	6
Nigussie et al. 2023 [32]	y	y	y	y	y	y	nc	y	y	8
Pardhan et al. 2024 [33]	y	y	y	y	y	y	nc	y	y	8
Paudel et al. 2023 [34]	y	y	y	y	y	y	nc	y	y	8
Palizgir et al. (2013) [35]	y	y	nc	nc	y	y	nc	y	y	6
Riise et al. (2025) [36]	y	y	nc	y	y	y	y	y	y	8
Rosas-matias et al. 2019 [37]	y	y	y	nc	y	y	nc	y	y	7
Salih et al. 2022 [38]	y	y	y	y	y	y	nc	y	y	8
Sayin et al. 2019 [39]	y	y	y	y	y	y	nc	y	y	8
Sharma et al. 2021 [40]	y	y	y	y	y	y	nc	y	y	8
Subramanian et al. 2024 [41]	y	nc	nc	y	y	y	y	y	y	7
Sun et al. 2016 [42]	y	y	y	y	y	y	nc	y	y	8
Tamene et al. 2026 [43]	y	y	y	y	y	y	nc	y	y	8
Tovila- zárate et al. 2012 [44]	y	y	y	nc	y	y	nc	y	y	7
Tripathi et al. 2026 [45]	y	nc	y	nc	y	y	nc	y	y	6

Item 1, Is the sample structure appropriate for representing the target population? Item 2, Were the study participants adequately recruited? Item 3, Was the sample size adequate? Item 4, Were the study subjects and settings described in detail? Item 5, Is the data analysis performed with sufficient coverage of the identified sample? Item 6, Were objective criteria and standards used to measure the condition? Item 7, Was the condition measured reliably? Item 8, Was an appropriate statistical analysis performed? Item 9, Are all confounding factors, subgroups, and significant differences identified and accounted for? *Y* yes, *N* no, *NC* not clear

### Prevalence of anxiety and depression in type 2 diabetes

**Fig. 2:**
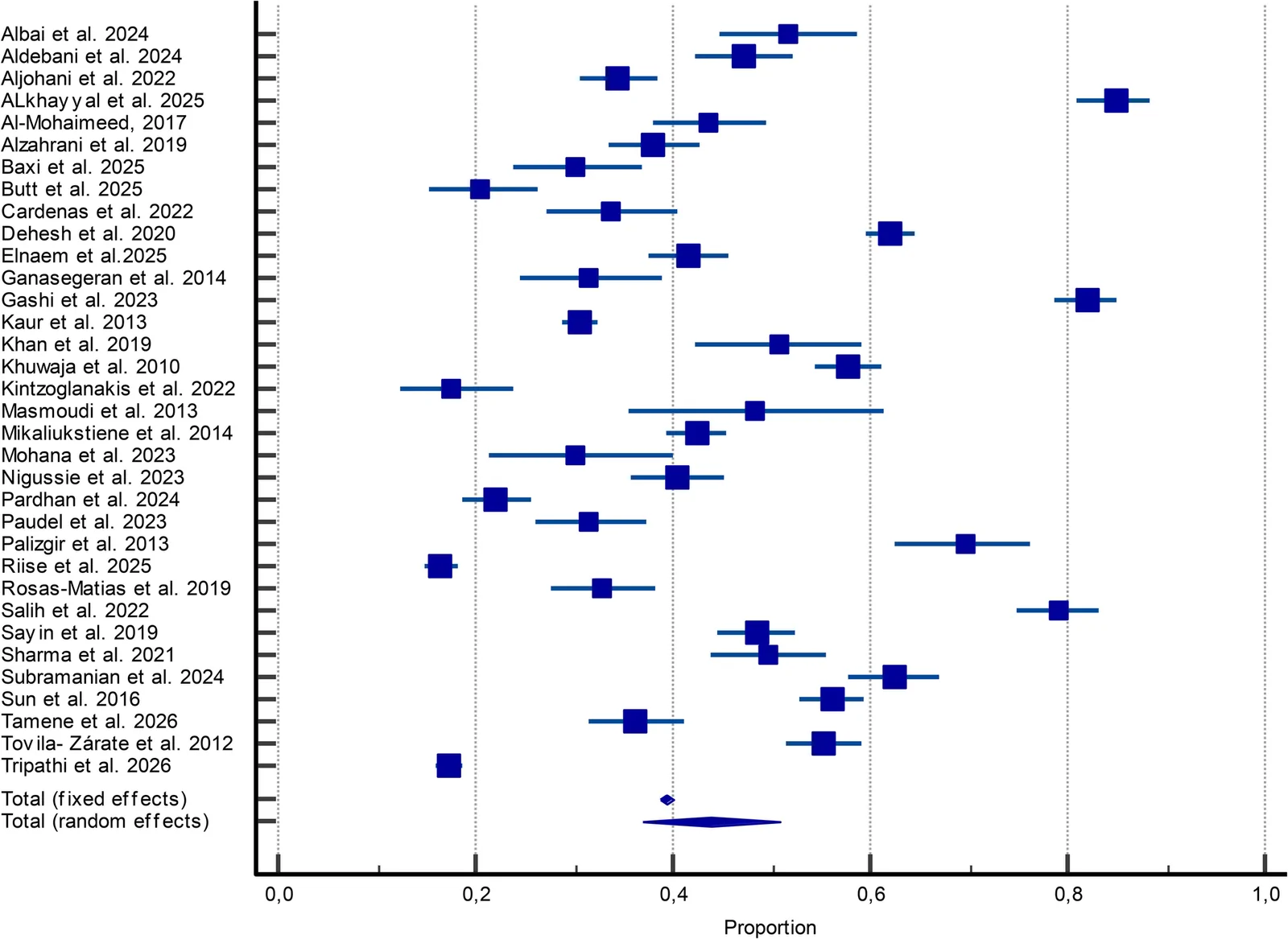
Forest plot of the pooled prevalence of anxiety symptoms among adults with type 2 diabetes (random-effects model). Squares represent the proportion (prevalence) reported by each individual study; horizontal lines represent 95% confidence intervals; square size is proportional to study weight in the random-effects model; the diamond represents the pooled prevalence estimate. I², heterogeneity statistic

**Fig. 3:**
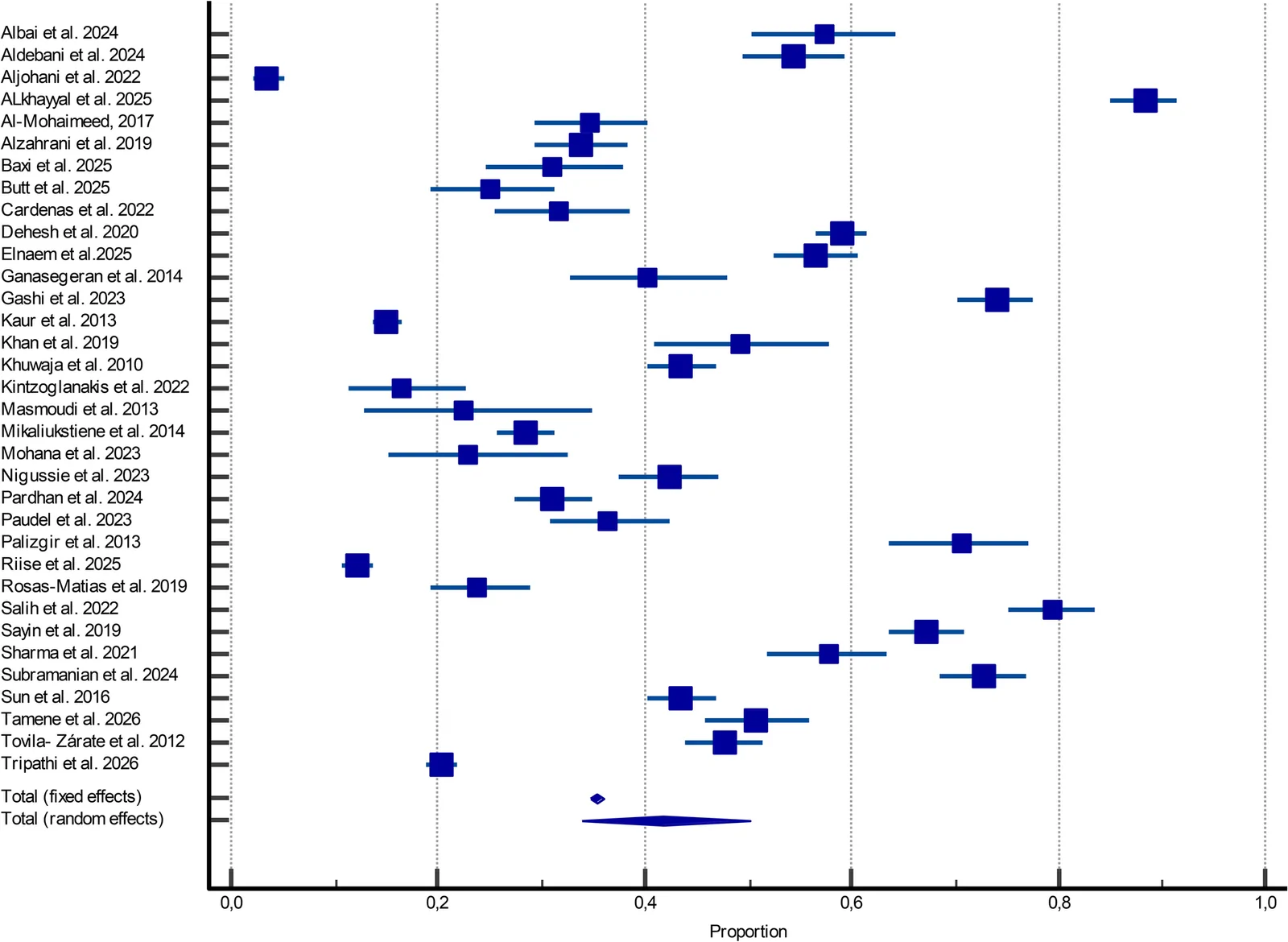
Forest plot of the pooled prevalence of depressive symptoms among adults with type 2 diabetes (random-effects model). Squares represent the proportion (prevalence) reported by each individual study; horizontal lines represent 95% confidence intervals; square size is proportional to study weight in the random-effects model; the diamond represents the pooled prevalence estimate. I², heterogeneity statistic

The meta-analysis included 34 studies assessing anxiety and depression and used a random-effects model (Figs. 2 and 3). The pooled prevalence among the populations evaluated in the included studies was 43.82% (95% CI: 36.86–50.90) for anxiety, with statistically significant heterogeneity across studies (I²: 99.06%; *P* < 0.0001), and 41.83% (95% CI: 33.83–50.06) for depression, with heterogeneity of I²: 99.31% (*P* < 0.0001). The complete data and funnel plots figures are provided in Supplementary Materials 3 (Anxiety) and 4 (depression). 

The forest plots (Figs. 2 and 3) show varying prevalence rates for anxiety and depression across the included studies, indicating considerable heterogeneity relative to the pooled prevalence.

The funnel plots for anxiety and depression, respectively (Supplementary Figs. 1 and 2), reveal asymmetry in the distribution of studies, suggesting possible publication bias. The Egger and Begg tests yielded different results across the outcomes. For anxiety and depression, the Egger test indicated statistically significant asymmetry (*p* = 0.0184); and (*p* = 0.0546), respectively.

#### Sensitivity analysis

The sensitivity analysis aimed to identify any outliers. It was conducted by removing studies one by one to determine whether the overall result changed. It was observed that individual studies did not influence the overall prevalence of anxiety and depression, which remained close to the global estimate (Table 3).

**Table 3:** Leave-one-out sensitivity analysis for the pooled prevalence of anxiety and depressive symptoms

Excluding	Anxiety				Depression			
	Combined prevalence and 95% CI	95% CI	I² (%)	*P*-value for heterogeneity	Combined prevalence and 95% CI	95% CI	I²	*P*-value for heterogeneity
Albai et al. 2024	43.58	36.51–50.79	99.08	< 0.0001	41.37	33.26–49.72	99.32	< 0.0001
Aldebani et al. 2024	43.72	36.60–50.97	99.08	< 0.0001	41.46	33.31–49.84	99.32	< 0.0001
Aljohani et al. 2022	44.11	36.94–51.41	99.08	< 0.0001	43.40	35.53–51.44	99.25	< 0.0001
ALkhayyal et al. 2025	42.47	35.77–49.31	98.97	< 0.0001	40.28	32.61–48.19	99.24	< 0.0001
Al-Mohaimeed, 2017	43.82	36.71–51.06	99.08	< 0.0001	42.05	33.85–50.47	99.33	< 0.0001
Alzahrani et al. 2019	44.00	36.85–51.27	99.08	< 0.0001	42.08	33.85–50.54	99.33	< 0.0001
Baxi et al. 2025	44.24	37.14–51.46	99.08	< 0.0001	42.16	33.99–50.56	99.33	< 0.0001
Butt et al. 2025	44.57	37.49–51.77	99.07	< 0.0001	42.36	34.18–50.76	99.33	< 0.0001
Cardenas et al. 2022	44.13	37.023–51.35	99.08	< 0.0001	42.36	34.18–50.76	99.33	< 0.0001
Dehesh et al. 2020	43.26	36.29–50.36	98.99	< 0.0001	41.31	33.22–49.64	99.27	< 0.0001
Elnaem et al.2025	43.89	36.71–51.19	99.08	< 0.0001	41.39	33.25–49.77	99.31	< 0.0001
Ganasegeran et al. 2014	44.20	37.10–51.41	99.08	< 0.0001	41.88	33.72–50.27	99.33	< 0.0001
Gashi et al. 2023	42.58	35.97–49.32	98.93	< 0.0001	40.84	32.96–48.95	99.27	< 0.0001
Kaur et al. 2013	44.23	36.82–51.78	99.06	< 0.0001	42.76	34.68–51.03	99.22	< 0.0001
Khan et al. 2019	43.61	36.54–50.82	99.08	< 0.0001	41.61	33.48–49.98	99.33	< 0.0001
Khuwaja et al. 2010	43.39	36.31–50.61	99.05	< 0.0001	41.78	33.49–50.31	99.32	< 0.0001
Kintzoglanakis et al. 2022	44.67	37.60–51.86	99.07	< 0.0001	42.67	34.50–51.04	99.32	< 0.0001
Masmoudi et al. 2013	43.69	36.63–50.88	99.08	< 0.0001	42.41	34.26–50.77	99.33	< 0.0001
Mikaliukstiene et al. 2014	43.86	36.62–51.24	99.08	< 0.0001	42.25	33.92–50.82	99.32	< 0.0001
Mohana et al. 2023	44.23	37.15–51.43	99.08	< 0.0001	42.41	34.25–50.79	99.33	< 0.0001
Nigussie et al. 2023	43.92	36.78–51.19	99.08	< 0.0001	41.82	33.61–50.25	99.33	< 0.0001
Pardhan et al. 2024	44.53	37.43–51.74	99.06	< 0.0001	42.17	33.91–50.66	99.33	< 0.0001
Paudel et al. 2023	44.20	37.09–51.44	99.08	< 0.0001	42.00	33.81–50.42	99.33	< 0.0001
Palizgir et al. 2013	43.04	36.04–50.18	99.07	< 0.0001	40.96	32.92–49.26	99.31	< 0.0001
Riise et al. 2025	44.76	37.96–51.65	98.90	< 0.0001	42.88	34.93–51.03	99.22	< 0.0001
Rosas-Matias et al. 2019	44.16	37.04–51.41	99.08	< 0.0001	42.41	34.21–50.82	99.32	< 0.0001
Salih et al. 2022	42.69	35.87–49.66	99.01	< 0.0001	40.65	32.78–48.77	99.28	< 0.0001
Sayin et al. 2019	43.68	36.53–50.96	99.08	< 0.0001	41.06	33.07–49.29	99.29	< 0.0001
Sharma et al. 2021	43.64	36.55–50.87	99.08	< 0.0001	41.35	33.24–49.71	99.32	< 0.0001
Subramanian et al. 2024	43.25	36.22–50.42	99.06	< 0.0001	40.88	32.93–49.07	99.29	< 0.0001
Sun et al. 2016	43.44	36.34–50.69	99.06	< 0.0001	41.78	33.49–50.31	99.30	< 0.0001
Tamene et al. 2026	44.05	36.91–51.32	99.08	< 0.0001	41.56	33.40–49.96	99.32	< 0.0001
Tovila- Zárate et al. 2012	43.47	36.36–50.71	99.06	< 0.0001	41.65	33.43–50.12	99.32	< 0.0001
Tripathi et al. 2026	44.72	38.04–51.51	98.82	< 0.0001	42.54	34.17–51.15	99.27	< 0.0001

*CI* confidence interval, *I*² heterogeneity statistic. Each row shows the pooled random-effects prevalence estimate obtained after excluding the named study from the analysis

A second sensitivity analysis was conducted using the three most commonly used instruments, subdivided by their respective cut-off points (Tables 4 and 5). Statistically significant differences in prevalence were observed. For the instruments assessing anxiety, estimates ranged from 29.30% (GAD-7 ≥ 10) to 60.05% (GAD-7 ≥ 5). Regarding the instruments assessing depression, notable prevalence rates were 66.53% for PHQ-9 ≥ 5 and 36.73% for PHQ-9 ≥ 10.

**Table 4:** Subgroup analysis by assessment instrument and cut-off point for anxiety symptoms

Instrument + cut-off point	Studies	Combined prevalence	95% CI	I² (%)	*P*-value for heterogeneity
HADS_≥10	3	50.84	46.04–55.63	0	0.9016
HADS_≥8	6	33.86	22.05–46.79	98.40	< 0.0001
GAD-7_≥5	4	60.05	45.02–74.17	97.25	< 0.0001
GAD-7_≥10	5	29.30	20.70-38.69	94.83	< 0.0001
DASS-21_≥8	4	45.69	19.84–72.86	99.48	< 0.0001
DASS-21_≥10	2	34.24	31.01–37.53	0	0.8583

*CI* confidence interval, *I*² heterogeneity statistic, *HADS* Hospital Anxiety and Depression Scale, *GAD-7* Generalized Anxiety Disorder-7, *DASS-21* Depression, Anxiety and Stress Scale-21

**Table 5:** Subgroup analysis by assessment instrument and cut-off point for depressive symptoms

Instrument + cutoff point	Studies	Combined prevalence	95% CI	I² (%)	*P*-value for heterogeneity
DASS-21 ≥ 10	6	27.94	9.43–51.64	99.53	< 0.0001
PHQ-9_≥5	4	66.53	54.21–77.80	96.07	< 0.0001
PHQ-9_≥10	6	36.73	25.52–48.73	97.02	< 0.0001
HADS_≥8	5	30.74	18.04–45.14	98.62	< 0.0001
HADS_≥10	3	43.47	26.47–61.29	92.00	< 0.0001

*CI* confidence interval, *I*² heterogeneity statistic, *DASS-21* Depression, Anxiety and Stress Scale-21, *PHQ-9* Patient Health Questionnaire-9

### Anxiety, depressive symptoms, and associated factors

#### Age

The association between age and mental health outcomes was explored in seventeen studies, with heterogeneous results [12, 15–19, 21–25, 27, 30, 32, 38, 40, 44], suggesting that age-related vulnerability may interact with disease duration, comorbidity burden, and self-management demands in T2DM. For depression, five studies identified a higher occurrence or a greater likelihood of the outcome among older individuals, including participants aged over 50, 57, and 70 years [12, 21, 23, 30, 32]. Conversely, two studies observed a lower probability of depression among older participants, demonstrating that advanced age may act as a protective factor [17, 22]. Alzahrani et al. (2019) and Elnaem (2025) observed that older age was inversely associated with depressive symptoms (OR = 0.95; 95% CI, 0.93–0.98; *p* < 0.001) and (adjusted OR = 0.41, 95% CI 0.25–0.68), although only one identified this protective effect in relation to anxiety [22]. At the same time, only one study reported a higher frequency of depression among younger individuals [19]. Regarding anxiety, only two studies found a higher occurrence among older participants, while one identified a lower likelihood in older people [23, 30]. In contrast, the majority of studies did not observe a statistically significant association between age and anxiety after statistical analysis; in 9 studies, no significant associations were found between age and either outcome.

#### Sex

Nineteen studies investigated the relationship between sex and the outcomes, with the majority showing no significant association [14, 15, 17, 18, 20, 23, 25, 26, 28–30, 32–36, 39, 44, 45]. Of these, eight identified a strong association between female sex and anxiety and depression. Most evidence pointed to greater vulnerability among women to psychological comorbidities in T2DM, which may reflect sex-related differences in diabetes burden, psychosocial stressors, and health-seeking behavior. Alzahrani et al. (2019) demonstrated that women had significantly higher odds of developing anxiety (OR = 1.79; 95% CI, 1.16–2.78) and depressive symptoms (OR = 2.69; 95% CI, 1.62–4.47) [17]. Similarly, Mikaliukštiene et al. [30] reported prevalences of 46.8% for anxiety and 32.3% for depressive symptoms in women (*p* < 0.001), while Palizgir et al. [35]confirmed that symptoms of both disorders were markedly more frequent in women, which corroborates other studies [28, 32]. Cardenas et al. [20] found that male sex was associated with a reduced risk of depression (OR 0.39 [95% CI, 0.18–0.81]; *P* = 0.01) and anxiety (OR 0.31 [95% CI, 0.16–0.65]; *P* = 0.002). In contrast to this trend, only the study by Riise et al. (2025) observed more prevalent depressive symptoms among men. However, the anxiety data in the same sample corroborated the female predominance observed in the other studies [36].

#### Body mass index (BMI)

Five studies investigated the relationship between BMI and anxiety and depression [13, 21, 22, 29, 30, 38]. The findings suggest an inconsistent association between BMI. Elnaem et al. [22]observed that Normal-weight patients had lower odds of presenting with depression and anxiety (adjusted OR = 0.44, 95% CI 0.27–0.72) compared to overweight patients. Aldebani’s study found that underweight patients exhibited higher anxiety scores compared to other groups (*p* = 0.006) [13]. Regarding depression, however, most studies did not find BMI to be statistically significant.

#### Complications

The association between chronic complications of T2DM and anxiety and depression was explored in ten studies [13, 18, 20, 21, 26, 29, 30, 34, 41, 44]. Overall, the articles demonstrated that the presence of complications influences the mental state of a person with type 2 diabetes. Nine studies identified a positive association between complications and the outcome of anxiety, while only six had a significant association with depression. Paudel et al. [34] It was observed that, after adjusting for confounding factors, multiple complications increased the odds of an individual developing anxiety and depression by 2.7-fold (OR 2.76, 95% CI 1.02–7.33) and 2.3-fold (adjusted OR 2.31, 95% CI 1.59–6.42), respectively; these findings are similar to those reported by Cárdenas (2022) for anxiety and depression (OR 2.96 [95% CI, 1.32–6.63]; *P* = 0.008) and for anxiety (OR 2.56 [95% CI, 1.20–5.48]; *P* = 0.01) [20]. Regarding specific complications, a study observed that painful neuropathy and nephropathy were associated with higher anxiety scores, whereas painful neuropathy and foot ulcers were associated with depression [26]. In another instance, the multivariate analysis identified a correlation between anxiety and the presence of nephropathy (*P* = 0.032), and between depression and peripheral neuropathy (*P* = 0.004) and retinopathy (*P* = 0.05) [29]. Thus, these results suggest that systemic involvement and clinical severity may act as risk factors for poorer mental health outcomes.

#### Social support

In the two studies that evaluated social support [16, 32] low social support was identified as a risk factor for anxiety and depression, with risk ranges of 2.15–5.35 and 2.35–6.62, respectively.

#### Duration of T2DM

The duration of T2DM was widely investigated across 16 studies, though the results were heterogeneous. Regarding depression, the majority of studies found that a longer disease duration increases the likelihood of an individual developing depressive symptoms, particularly in those diagnosed more than ten years prior or as the duration of the condition progressively increases [12, 15, 17, 18, 30, 33, 37, 42]. Alzahrani et al. [17] It was found that a one-unit increase in the time elapsed since diagnosis contributes to a 1.07-fold increase in the likelihood of having depression (95% CI: 1.02–1.11). In another study, the analysis found that disease duration exceeding 10 years increased the odds of depression by 2.18 times compared with those with a duration of less than 5 years (adjusted OR = 2.18; 95% CI: 1.16–4.11) [42]. In contrast, studies indicate a higher risk of depression in patients with a recent diagnosis [22, 38]. Supporting these findings, a study of 300 adults with T2DM conducted at a diabetes center showed that patients with diabetes for 10 to 20 years are less likely (adjusted OR 0.16; 95% CI: 0.06–0.44) to suffer from depression and anxiety than those with diabetes for 10 years or less; our results also show that patients with diabetes for 10 to 20 years are less likely to exhibit anxiety (adjusted OR 0.39; 95% CI: 0.2–0.76) than those with diabetes for 10 years or less [16]. Regarding anxiety, although studies have linked its development to individuals with a longer duration of the disease [37] and others in more recent diagnoses [16, 22]. Most studies did not find a significant association between disease duration and anxiety symptoms [12, 14, 15, 19, 38, 45].

#### Glycemic control

Glycemic control, assessed using glycated hemoglobin (HbA1c), was evaluated in eight studies [12, 16–18, 24, 25, 29, 45]. For anxiety and depression, four studies identified inadequate glucose management as a risk factor [12, 17, 18, 24, 29]. In Albai’s study [12], HbA1c level above 8.5% (sensitivity 45.8, specificity 83.1, AUROC = 0.64, *p* < 0.0001) predicts, respectively, a higher incidence of anxiety and depression in these patients. In another study, the presence of both conditions was significantly associated with inadequate glycemic control (*p* = 0.007 for depression and *p* = 0.011 for anxiety) [18]. Furthermore, some studies found no association between glycemic control and either outcome [16, 25, 45]. Overall, these articles indicate that the literature is not unanimous but points to a negative relationship between the presence of both outcomes and altered glycemic control.

#### Income

The income factor was analyzed in eleven studies [13, 15, 16, 20, 23, 25, 28, 29, 32, 33, 40]; a significant association with the anxiety outcome was found in only two [13, 28], and only four [13, 20, 23, 28] managed to correlate income with the outcome. Overall, patients with lower income had higher anxiety and depression scores compared to individuals with higher income [23, 28]. Another study identified a link between higher income and a reduced risk of anxiety and depression [20].

#### Level of education

Of the thirteen studies that assessed educational level, six found a significant association with anxiety and depression [13, 15, 16, 20, 21, 23–25, 30, 32, 33, 44, 45]. Higher levels of anxiety and depression were more frequently reported among individuals with lower levels of education [15, 20, 23, 30, 33, 45]. A study by Gashi (2023) involving 596 adults with type 2 diabetes (T2DM) receiving primary care found that individuals with only a primary education were 1.68 times more likely to develop depression compared to those with higher levels of education (OR = 1.69; 95% CI: 1.15–2.48) [24], a finding that corroborates data from other studies [15, 23, 33]. A cross-sectional study of 209 individuals with T2DM treated at two tertiary care hospitals identified higher educational attainment as a protective factor against anxiety (OR 0.47, 95% CI, 0.25–0.90; *P* = 0.02) and depression (OR 0.23, 95% CI, 0.11–0.46; *P* < 0.001) in this population [20].

#### Marital status

Fourteen studies evaluated marital status [13, 15, 16, 19, 21, 23–25, 29, 32, 33, 41, 44, 45]. Among them, no significant association was found in 13 regarding the outcome of depression, nor in 11 regarding anxiety.

## Discussion

This systematic review with meta-analysis found a pooled prevalence of 43.82% for anxiety symptoms and 41.83% for depressive symptoms in adults with T2DM. However, these estimates must be interpreted in light of the significant heterogeneity observed across studies, with I² values exceeding 99% for both outcomes. In prevalence meta-analyses, high I² values are common and should not be interpreted in isolation; understanding heterogeneity requires considering the distribution of estimates, population characteristics, and sensitivity and subgroup analyses [11, 46]. Indeed, a recent umbrella review found pooled prevalence rates of depression in T2DM ranging from 8.9% to 61.6%, with I² values between 89.41% and 99.96%, and identified differences by region and assessment method [9]. Similarly, a recent meta-analysis on anxiety in individuals with T2DM found an I² of 99.14% and demonstrated variation in estimates depending on the instrument used [10]. These findings reinforce that a single pooled estimate should not be interpreted as a universal prevalence applicable to all populations with T2DM. A meta-analysis found a prevalence of depressive symptoms of 25.9% (95% CI: 20.6–31.6%) among patients with T2DM in the Chinese population [47]. In another study that assessed the prevalence of anxiety disorders in people with T2DM from different countries, a prevalence of 18% was observed [48]. Although these reviews report lower prevalences than those reported in this meta-analysis, the values fall within the range observed here.

In studies that simultaneously assessed both outcomes, anxiety symptoms were more prevalent than depressive symptoms. From a methodological standpoint, this difference should be interpreted with caution. Wide confidence intervals, their overlap, and high heterogeneity across studies make it impossible to conclude, based solely on these estimates, that anxiety symptoms are more prevalent than depressive symptoms in people with T2DM. Furthermore, comparisons with studies assessing diagnosed anxiety disorders must distinguish between clinical diagnoses and symptoms identified via screening tools. The American Diabetes Association emphasizes that symptoms of anxiety and depression, diabetes distress, and psychiatric disorders represent related yet distinct constructs, and that a positive screening result requires further clinical evaluation when indicated [49].

From a clinical perspective, this pattern may reflect differences in symptom thresholds, instrument sensitivity, and the contextual burden associated with chronic disease self-management, including the constant demands of glucose monitoring and complication prevention [6, 17, 36, 48]. A relevant example is the research conducted in the Norwegian population [36], which showed a predominance of anxiety symptoms compared to depressive symptoms. This may reflect the chronic and demanding nature of T2DM, which requires ongoing monitoring of diet, pharmacotherapy, and glycemic control. The need to prevent acute and chronic complications may place individuals in a state of persistent vigilance, leading to anxiety symptoms as an important emotional response to the burden of self-management of the disease [6, 36].

Evidence suggests a bidirectional relationship between T2DM and anxiety and depressive symptoms, although the cross-sectional design of most included studies limits causal inference [50, 51]. The literature suggests shared mechanisms linking anxiety, depressive symptoms, and T2DM, including inflammatory and neuroendocrine pathways that may also contribute to poorer metabolic control [52, 53]. Alterations in cerebral glucose metabolism, hormonal dysregulation, inflammatory processes, and oxidative stress may represent interconnected pathways linking T2DM and mental health outcomes; these mechanisms remain inferential in the studies included here [52–55]. Inflammation may partially mediate the bidirectional relationship between these outcomes, particularly through chronic low-grade inflammatory activation associated with poor metabolic control [51, 55]. Stuart et al. (2012) point out that hyperglycemia and hyperinsulinemia, characteristic of T2DM, induce a systemic pro-inflammatory state, which can trigger depressive symptoms [55]. Depressive symptoms may activate the hypothalamic-pituitary-adrenal (HPA) axis, increase cortisol secretion, and thereby worsen insulin resistance and glycemic control [54, 55]. Although such mechanisms are plausible, further studies are needed to explore these common pathways in an integrated way.

The chronic nature of T2DM and the need for sustained self-management impose a substantial psychosocial burden that may contribute to emotional exhaustion, vigilance, and reduced perceived quality of life [56, 57]. The need for constant monitoring and the fear of acute and chronic complications can lead to a negative self-perception of health [58]. This continuous demand can generate emotional fatigue, persistent vigilance, and a sense of loss of autonomy, fostering hopelessness and demotivation when sustained over time [57, 59]. Furthermore, anxiety symptoms arise not only as an immediate response but also as a reflection of the continuous emotional strain caused by the loss of predictability of physical well-being [6]. From this perspective, anxiety and depressive symptoms may negatively affect T2DM by reducing treatment adherence, worsening self-care behaviors, contributing to glycemic dysregulation, and increasing the risk of diabetes-related complications [60]. Poor glycemic control, in turn, may generate feelings of failure and guilt, which may contribute to anxiety and depressive symptoms [12]. This is a cycle in which both conditions reinforce each other and manifest themselves in ways that impair treatment and quality of life [59].

In this review, women were the sociodemographic factor most consistently associated with a higher prevalence of anxiety and depressive symptoms. However, the direction and magnitude of the association varied across studies. This finding corroborates previous epidemiological evidence pointing to a predominance of mood disorders among women in the context of diabetes [61]. This pattern is likely multifactorial, involving hormonal, physiological, and sociocultural factors [62]. In T2DM, which requires rigorous self-management and persistent lifestyle changes, the accumulation of these responsibilities can hinder adaptation to disease management, worsen glycemic control, and increase psychological distress [63]. In particular, although some studies have shown a higher prevalence of anxiety or depression symptoms among women, the majority of studies evaluating sex did not identify a statistically significant association. Thus, female sex should be interpreted as a possible correlate of greater psychological vulnerability in certain contexts, rather than as a consistent determinant across all populations with T2DM.

Among the clinical factors evaluated, complications related to T2DM showed a relatively consistent association with psychological outcomes in a significant proportion of the included studies. This relationship is supported by external longitudinal evidence. In a prospective registry-based study following 265,799 adults with T2DM, the presence or development of any diabetic complication was associated with a higher subsequent risk of depression and/or anxiety (HR = 1.77; 95% CI 1.73–1.80) [64]. The risk was elevated across all evaluated categories of complications, with the strongest association observed for lower-limb amputation [64]. These data complement the predominantly cross-sectional findings of the present review and suggest that a greater clinical burden of the disease may contribute to increased psychological vulnerability. However, the underlying mechanisms are likely multifactorial.

Although only two studies included in this review directly assessed social support, both indicated that lower social support was associated with a higher occurrence of psychological symptoms. This association is supported by a specific meta-analysis of individuals with T2DM, involving 11 studies and 3,151 participants. In that analysis, individuals with lower social support had approximately twice the odds of depression compared to those with higher social support (OR = 2.02; 95% CI 1.51–2.70) [65]. However, given the small number of studies on social support included in the present review, this factor should be interpreted as clinically relevant and biologically plausible, yet not as one of the factors most consistently demonstrated across the body of studies analyzed here.

Findings on glycemic control were heterogeneous: some studies reported an association between higher HbA1c levels and psychological symptoms, whereas others found no statistically significant relationship. Recent evidence reinforces the need for such a cautious interpretation. A global systematic review and meta-analysis published in 2026 found mean HbA1c levels to be approximately 0.30% points higher in individuals with T2DM and depression compared to those without depression [66]. However, the same meta-analysis found no statistically significant association when inadequate glycemic control was analyzed as a categorical outcome; furthermore, it rated the certainty of the evidence as very low and identified high heterogeneity [66]. Thus, current data support an association between mental health and glycemic control, but do not justify interpreting changes in HbA1c as an independent or uniform predictor of anxiety and depression.

However, beyond the pathophysiological and behavioral mechanisms involved, interpretation of these prevalences is hampered by methodological heterogeneity, including variation in instruments, cutoffs, study settings, and participant selection [11]. Variability in the instruments used may have compromised comparability across studies, as each study uses a different cutoff point. The methodological quality of the studies themselves may act as a factor responsible for the different prevalences observed.

The leave-one-out sensitivity analysis showed that the pooled prevalence estimates remained largely unchanged after sequentially excluding individual studies, indicating that no single study disproportionately influenced the overall results. This finding strengthens confidence in the robustness of the pooled estimates despite the substantial between-study heterogeneity.

One of the most significant sources of heterogeneity observed in this review was the use of different instruments and cut-off points. In stratified analyses, the PHQ-9 with a cut-off of ≥ 5 yielded a pooled estimate of depressive symptoms substantially higher than that obtained with a cut-off of ≥ 10. A similar pattern was observed for the GAD-7, with estimates also varying according to the adopted threshold. These results do not, in isolation, demonstrate that the cut-off point is solely responsible for the heterogeneity, given that study groups also differ in clinical, social, and geographic characteristics; however, they highlight that the operational definition of the outcome directly affects comparability across studies. A recent umbrella review identified differences in depression prevalence across measurement approaches [9] a meta-analysis of anxiety in people with T2DM also found differences between studies employing distinct screening instruments [10]. Therefore, the standardization of instruments and the explicit reporting of cut-off points are crucial for improving the comparability of future prevalence estimates.

Understanding the relationship between T2DM and mental health suggests that effective glycemic management is closely linked to the identification and management of anxiety and depressive symptoms in routine diabetes care. However, the direction of influence is likely bidirectional [51]. These conditions are frequently underrecognized in routine diabetes care, representing a clinical challenge and potentially contributing to inadequate disease management, poorer metabolic control, and worse outcomes [6, 7, 12]. In this context, it is necessary to consider actions that can improve clinical outcomes. The findings support implementing screening protocols with validated instruments soon after T2DM diagnosis, along with regular psychosocial follow-up within multidisciplinary diabetes care [49]. Furthermore, this support should extend to family members, who can identify systemic factors that may interfere with the patient’s self-management and adherence to treatment [5].

The findings of this review should be interpreted in light of certain limitations. First, the included studies predominantly employed a cross-sectional design, making it impossible to establish temporality or causal relationships between the associated factors and symptoms of anxiety and depression. Second, extremely high heterogeneity was observed among the studies, reflecting differences in populations, recruitment settings, clinical characteristics, and especially the instruments and cut-off points used. In prevalence meta-analyses, very high I² values are common, and the pooled estimate should be understood as an average across different contexts rather than a universal value [46]. Furthermore, many studies used screening instruments; thus, the estimated prevalence rates predominantly represent clinically relevant symptoms rather than necessarily confirmed psychiatric diagnoses. The predominance of studies conducted in specific geographic regions and hospital settings may also limit the generalizability of the findings. Although the leave-one-out analysis showed that no single study substantially altered the pooled estimate, this result does not rule out significant heterogeneity among the studies. Finally, the asymmetry observed in the funnel plots should be interpreted with caution, as it may reflect not only publication bias but also small-study effects, clinical and methodological heterogeneity, or other systematic differences between the studies. Furthermore, this review was based primarily on cross-sectional studies, which preclude drawing causal inferences.

Key strengths of this review include the inclusion of 34 studies from diverse regions, the use of five databases, the performance of meta-analyses for both psychological outcomes, the exploration of potential sources of heterogeneity, and the examination of associations between the meta-analytic results and potential predictors of anxiety and depression. A leave-one-out analysis demonstrated the stability of the pooled estimates upon the sequential exclusion of studies. In contrast, analyses stratified by assessment instrument and cutoff point highlighted the importance of methodological choices for interpreting prevalence rates. Furthermore, the synthesis of associated clinical and psychosocial factors extends the interpretation beyond mere prevalence estimates and helps identify priority areas for future research [48, 61]. These aspects represent a significant contribution, particularly given the extensive methodological variability documented in recent literature on mental health and T2DM [9].

Our findings have the potential to guide actions and public policies that promote integrated diabetes and mental health care, especially regarding screening, early detection, and coordinated follow-up for people with T2DM.

## Conclusion

Anxiety and depressive symptoms are common among adults with T2DM, with pooled prevalence rates of 43.82% and 41.83%, respectively. However, the high heterogeneity across studies indicates that these estimates should not be interpreted as universal values, as they vary according to population characteristics, clinical settings, assessment tools, and the cut-off points used. Overall, the associated sociodemographic and clinical factors exhibit varying levels of consistency. In the descriptive synthesis of the studies, the presence of chronic diabetes complications, low social support, female gender, and lower levels of education are associated with a higher frequency of anxiety and depression. Conversely, age, body mass index, duration of diabetes, income, and marital status were factors that, for the most part, proved non-significant for both outcomes. Inadequate glycemic control, despite inconsistent findings, was associated with both outcomes in part of the available evidence. This magnitude reflects variability across studies, suggesting that the results are influenced by methodological and clinical factors, as well as the context in which the individual with T2DM lives. Therefore, there is a reinforced need for diabetes care strategies that incorporate routine screening for anxiety and depressive symptoms, particularly among patients with suboptimal metabolic control, treatment non-adherence, or established complications.

## Supplementary Information

Below is the link to the electronic supplementary material.

Supplementary Material 1

Supplementary Material 2

Supplementary Material 3

Supplementary Material 4

## Data Availability

No datasets were generated or analysed during the current study.

## Declarations

This study was conducted in accordance with the principles of the Declaration of Helsinki. However, as this is a systematic review of previously published studies, ethical approval and informed consent were not required. No institutional review board approval was required because the study did not directly involve human participants.
