# Supplementary material for: Pooled prevalence of anxiety and depressive symptoms among adults with type 2 diabetes: a systematic review and meta-analysis
Source: J Diabetes Metab Disord. 2026 Sep 24;25(2):265. doi: 10.1007/s40200-026-02079-z (PMC13612785; doi:10.1007/s40200-026-02079-z)
Supplement: Supplementary file 1 [file 40200_2026_2079_MOESM1_ESM.docx]

**Search strategies**

**Supplementary Material 1 Database-specific search strategies (PubMed, Embase, Scopus, Web of Science, and PsycINFO)**

**Pubmed:**

**Search date:** July 6, 2026

**Number of records retrieved:** 2,486

Three blocks of search strategies were created and subsequently combined:

**#1 population):**"Diabetes Mellitus, Type 2"[Mesh] OR "type 2 diabetes"[tiab] OR "type 2 diabetic"[tiab] OR "type II diabetes"[tiab] OR "T2DM"[tiab] OR "NIDDM"[tiab] OR "non-insulin dependent diabetes"[tiab]

**#2 outcome:**"Depression"[Mesh] OR "Depressive Disorder"[Mesh] OR "Anxiety"[Mesh] OR "Anxiety Disorders"[Mesh] OR "depression"[tiab] OR "depressive"[tiab] OR "anxiety"[tiab] OR "mood disorder"[tiab] OR "mood disorders"[tiab] OR "psychological distress"[tiab] OR "diabetes distress"[tiab]

**#3 context:** "Prevalence"[Mesh] OR "Cross-Sectional Studies"[Mesh] OR "prevalence"[tiab] OR epidemiolog*[tiab] OR "cross-sectional"[tiab] OR "cross sectional"[tiab]

**#4 Combined search**: #1 AND #2 AND #3

**Final search (after excluding publication types)**

#4 NOT ("review"[pt] OR "systematic review"[pt] OR "meta-analysis"[pt] OR "editorial"[pt] OR "letter"[pt] OR "comment"[pt] OR "case reports"[pt])

**Embase:**

**Search date:** July 6, 2026

**Number of records retrieved:** 4,565

Three blocks of search strategies were created and subsequently combined:

**#1 (population):** 'non insulin dependent diabetes mellitus'/exp OR 'type 2 diabetes':ti,ab,kw OR 'type 2 diabetic':ti,ab,kw OR 'T2DM':ti,ab,kw OR 'NIDDM':ti,ab,kw

**#2 (outcome):** 'depression'/exp OR 'anxiety'/exp OR 'anxiety disorder'/exp OR 'depression':ti,ab,kw OR 'depressive':ti,ab,kw OR 'anxiety':ti,ab,kw OR 'mood disorder':ti,ab,kw OR 'psychological distress':ti,ab,kw OR 'diabetes distress':ti,ab,kw

**#3 (context):** 'prevalence'/exp OR 'cross-sectional study'/exp OR 'prevalence':ti,ab,kw OR 'epidemiolog*':ti,ab,kw OR 'cross-sectional':ti,ab,kw

**Combined blocks**: #1 AND #2 AND #3 NOT ('conference abstract'/it OR 'conference paper'/it)

**SCOPUS**

**Search date:** July 6, 2026

**Number of records retrieved:** 4,913

( TITLE-ABS-KEY ( "type 2 diabetes" OR "type 2 diabetic" OR "type II diabetes" OR "T2DM" OR "NIDDM" OR "non-insulin dependent diabetes" ) ) AND ( TITLE-ABS-KEY ( "depression" OR "depressive" OR "anxiety" OR "mood disorder" OR "psychological distress" OR "diabetes distress" ) ) AND ( TITLE-ABS-KEY ( "prevalence" OR "epidemiolog*" OR "cross-sectional" ) ) AND ( LIMIT-TO ( DOCTYPE , "ar" ) )

**WEB OF SCIENCE:**

**Search date:** July 6, 2026

**Number of records retrieved:** 3,407

The refinement was performed by selecting the "articles only" option in the filter on the left, and the file was split into four exports.

TS=("type 2 diabetes" OR "type 2 diabetic" OR "type II diabetes" OR "T2DM" OR "NIDDM" OR "non-insulin dependent diabetes") AND TS=("depression" OR "depressive" OR "anxiety" OR "mood disorder" OR "psychological distress" OR "diabetes distress") AND TS=("prevalence" OR "epidemiolog*" OR "cross-sectional")

**PSYCINFO:**

**Search date:** July 6, 2026

**Number of records retrieved**: 95

Three blocks of search strategies were created and subsequently combined:

**#1 (population):"**type 2 diabetes" OR "type 2 diabetic" OR "type II diabetes" OR T2DM OR NIDDM OR "non-insulin dependent diabetes"

**#2 (outcome):** depression OR depressive OR anxiety OR "mood disorder" OR "psychological distress" OR "diabetes distress"

**#3 (context):** prevalence OR epidemiolog* OR "cross-sectional"

**Combined blocks:** Line #1 AND Line #2 AND Line #3

Search blocks were combined using the Boolean operator AND; synonymous terms within each block were combined using OR. [Mesh]/[tiab] and /exp,:ti,ab,kw denote controlled-vocabulary and free-text field tags, respectively, specific to each database interface.
