# Supplementary material for: Pooled prevalence of anxiety and depressive symptoms among adults with type 2 diabetes: a systematic review and meta-analysis
Source: J Diabetes Metab Disord. 2026 Sep 24;25(2):265. doi: 10.1007/s40200-026-02079-z (PMC13612785; doi:10.1007/s40200-026-02079-z)
Supplement: Supplementary file 2 [file 40200_2026_2079_MOESM2_ESM.docx]

**Supplementary Table 2 - Characteristics of the included studies (n=34)**

| **Studies** | **Year of the research** | **Country** | **Region** | **Design** | **Scenario** | **Participants (Average age and/or Median + SD)** | **Sample** | **n (%) women** | **Scale Used**  **Anxiety** | **Cutoff point** | **Prevalence n (%) Anxiety** | **Scale Used**  **Depression** | **Cutoff point** | **Prevalence n (%) Depression** |
| --- | --- | --- | --- | --- | --- | --- | --- | --- | --- | --- | --- | --- | --- | --- |
| Albai et al. 2024[1] | 2024 | Romania | Europe | Cross-sectional | Hospital | 66 (median) | 209 | 136 (65.07) | HADS | **≥** 10 | 108 (51.7) | HADS | **≥** 10 | 120 (57.5) |
| Aldebani et al. 2024[2] | ND | Saudi Arabia | Asia | Cross-sectional | Family Health Center | 47 (median) | 412 | 205 (42.5) | GAD-7 | **≥** 5 | 194 (47) | PHQ-9 | **≥** 5 | 224 (54.4) |
| Aljohani et al. 2022[3] | 2020 | Saudi Arabia | Asia | Cross-sectional | Primary Health Care Center | ND | 602 | 392 (65.1) | DASS-21 | **≥** 10 | 207 (34.4) | DASS-21 | **≥** 10 | 20 (3.3) |
| Alkhayyal et al. 2025[4] | 2024-2025 | Saudi Arabia | Asia | Cross-sectional | Patients residing in the eastern region of Saudi Arabia | ND | 391 | 129 (33.0) | GAD-7 | ND | 332 (84.91) | PHQ-9 | **≥** 5 | 346 (88.49) |
| Al-Mohaimeed, 2017[5] | 2016 | Saudi Arabia | Asia | Cross-sectional | Diabetes Center at King Saud Hospital | ND | 300 | 150 (50) | HADS | **≥** 8 | 131 (43.6% 95% CI: 37.9-49.3%) | HADS | **≥** 8 | 104 (34.8% CI 95%: 29-40%) |
| Alzahrani et al. 2019[6] | ND | Saudi Arabia | Asia | Cross-sectional | primary health centers  Ministry of Affairs  National Guard Health  (MNGHA) | 56.9 ±11.1 | 450 | 194 (43.1) | DASS-21 | **≥** 8 | 171 (38) | DASS-21 | **≥** 10 | 152 (33.8) |
| Baxi et al. 2025[7] | 2025 | India | Asia | Cross-sectional | Hospital | 54.6 ± 9.2 | 200 | 92 (46) | GAD-7 | **≥** 10 | 60 (30) | PHQ-9 | **≥** 10 | 62 (31) |
| Butt et al. 2025[8] | 2024 | Pakistan | Asia | Cross-sectional | Hospital | 53.7±13.0 | 216 | 132 (61.1) | HADS | **≥** 11 | 44 (20.4) | HADS | **≥** 11 | 54 (25) |
| Cardenas et al. 2022[9] | 2020 | ND | ND | Cross-sectional | Hospital | 69.8 ± 9.6 | 208 | 120 (57.7) | DASS-21 | ≥10 | 70 (33.7% 95% CI 27.3-40.5) | DASS-21 | ≥10 | 66 (31.7% 95% CI 25.4-38.5%) |
| Dehesh et al. 2020[10] | 2018 | Iran | Asia | Cross-sectional | ND | 47.12 ± 12.52 | 1500 | 770 (51.3) | HAM-A | ≥14 | 930 (62% 95% CI: 59.51-66.27) | BDI | ≥18 | 885 (59% 95% CI: 54.48-63.12) |
| Elnaem et al. 2025[11] | 2023 and 2023 | Indonesia and Malaysia | Asia | Cross-sectional | ND | ND | 606 | 391 (64.5) | GAD-7 | **≥** 10 | 252 (41.6) | PHQ-9 | **≥** 10 | 343 (56.6) |
| Ganasegeran et al. 2014[12] | 2013 | Malaysia | Asia | Cross-sectional | Hospital | 36.9 ±15.9 | 169 | 70 (41.4) | HADS | **≥8** | 53 (31.4) | HADS | **≥8** | 68 (40.3) |
| Gashi et al. 2023[13] | 2022 to 2023 | Kosovo | Europe | Cross-sectional | Primary Health Care | ND | 596 | 266 (44.6) | DASS-21 | **≥8** | 489 (82) | DASS-21 | **≥10** | 441 (74) |
| Kaur et al. 2013[14] | ND | Malaysia | Asia | Cross-sectional | Primary Care | ND | 2508 | 1533 (61.1) | DASS-21 | **≥8** | 768 (30.5) | DASS-21 | **≥10** | 376 (15) |
| Khan et al. 2019[15] | 2018 | Pakistan | Asia | Cross-sectional | Hospital | 57.01 ± 11.23 | 142 | 81 (57) | HADS | **≥10** | 72 (50.7) | HADS | **≥10** | 70 (49.2) |
| Khuwaja et al. 2010[16] | ND | Pakistan | Asia | Cross-sectional | Outpatient Clinics | ND | 889 | 511 (57.5) | HADS | ND | 514  (57.9 CI 95%: 54.7 - 61.2) | HADS | ND | 387  (43.5 CI 95%: 40.3 - 46.8) |
| Kintzoglanakis et al. 2022[17] | 2019-2020 | Greece | Europe | Cross-sectional | Urban Primary Care | 68.04 (SD 9.23) | 182 | 81 (43.4) | GAD-7 | **≥** 10 | 32 (17.7) | PHQ-9 | **≥** 10 | 30 (16.6) |
| Masmoudi et al. 2013[18] | 2010 | Tunisia | Africa | Cross-sectional | Hospital | 54.5 (9.4) | 62 | 46 (74.2) | HADS | **≥** 10 | 25 (40.3) | HADS | **≥** 10 | 14 (22.6) |
| Mikaliukstiene et al. 2014[19] | ND | Lithuania | Europe | Cross-sectional | ND | 59.0 | 1022 | 650 (63.6) | HADS | **≥** 8 | 433 (42.4) | HADS | **≥** 8 | 291 (28.5) |
| Mohana et al. 2023[20] | ND | Somalia | Africa | Cross-sectional | Hospital | ND | 100 | 28 (28) | DASS-21 | **≥** 8 | 30 (30) | DASS-21 | **≥** 10 | 23 (23) |
| Nigussie et al. 2023[21] | 2022 | Ethiopia | Africa | Cross-sectional | Hospital | 53.5 years with an interquartile range of 45 to 62 years. | 416 | 198 (47.5) | HADS | **≥** 8 | 168 (40.4) | HADS | **≥** 8 | 176 (42.3) |
| Pardhan et al. 2024[22] | 2022 | Bangladesh | Asia | Cross-sectional | Home visits | 52.70 ± 11.56 | 600 | 329 (54.83) | GAD-7 | **≥** 10 | 132 (22) | PHQ-9 | **≥** 10 | 186 (31) |
| Paudel et al. 2023[23] | 2021 | Nepal | Asia | Cross-sectional | Health Units | 56.17 ± 11.81 | 283 | 122 (43.1) | HADS | **≥** 8 | 89 (31.4) | PHQ-9 | **≥** 10 | 103 (36.4) |
| Palizgir et al. 2013[24] | 2012 | Iran | Asia | Cross-sectional | Hospital | ND | 184 | 117 (63.6) | BAI-1 | **≥** 14 | 128 (69.6) | BDI-II | **≥** 14 | 130 (70.7) |
| Riise et al. 2025[25] | ND | Norway | Europe | Cross-sectional | Participants in a HUNT 4 survey | 67.3  (31.3-94.7) | 1954 | 836 (42.8) | HADS-A | **≥** 8 | 320 (16.4) | HADS-D | **≥** 8 | 238 (12.2) |
| Rosas-Matias et al. 2019[26] | 2019 | Peru | America | Cross-sectional | Hospital | ND | 327 | 231 70.64 | EAL - 20 | **≥** 10 | 107 (32.72) | EPD-6 | **≥** 8 | 78 (23.85) |
| Salih et al. 2022[27] | 2021-2022 | Iraq | Asia | Cross-sectional | Diabetes Center | 55.47 ± 8.35 | 380 | 172 (45.3) | GAD-7 | **≥** 5 | 301 (79.2) | PHQ-9 | **≥** 5 | 302 (79.5) |
| Sayin et al. 2019[28] | 2017-2018 | Turkey | Asia | Cross-sectional | Hospital | 59.98±11.66 | 636 | 381 (59.9) | HADS-A | **≥** 10 | 308 (48.4) | HASD-D | **≥** 7 | 428 (67.3) |
| Sharma et al. 2021[29] | 2018-2019 | Nepal | Asia | Cross-sectional | Hospital | 59.50 ± 11.72 | 296 | 178 (59.5) | GAD-7 | **≥** 5 | 147 (49.7) | PHQ-9 | **≥** 5 | 171 (57.8) |
| Subramanian et al. 2024[30] | 2023 | India | Asia | Cross-sectional | Hospital | 38 ± 6 | 450 | 224 (49.8) | GAD-7 | **≥** 5 | 281(62.4) | PHQ-9 | **≥** 5 | 328 (72.9) |
| Sun et al. 2016[31] | ND | China | Asia | Cross-sectional | Community Clinics | 63.9 ± 10.2 | 893 | 523 (58.6) | Zung Self-Rating Anxiety Scale | **≥** 50 | 501 (56.1) | Zung Depression Self-Rating Scale | **≥** 50 | 389 (43.6) |
| Tamene et al. 2026[32] | 2023 | Ethiopia | Africa | Cross-sectional | Hospital | 56.89 ± 16.1 | 409 | 237 (57.9) | GAD-7 | **≥10** | 148 (36.2) | PHQ-9 | **≥** 10 | 208 (50.9) |
| Tovila-Zárate et al. 2012[33] | 2011 | Mexico | America | Cross-sectional | Hospital | 47.39 ± 12.79 | 702 | 392 (55.8) | HAM-A | **ND** | 388 (55.10% 95% CI: 51.44-58.93) | HAM-D | **≥14** | 335 (48.27% 95% CI: 44.48-52.06) |
| Tripathi et al. 2026[34] | 2022 | India | Asia | Cross-sectional | Clinic | 49.7 ± 10.2 | 2730 | 942 (34.5) | GAD-7 | **ND** | 472 (17.3) | PHQ-9 | **ND** | 554 (20.3) |

n, sample size; ND, not available; DASS-21, Depression, Anxiety and Stress Scale; PHQ-9, Patient Health Questionnaire-9; HADS, Hospital Anxiety and Depression Scale; GAD-7, Generalized Anxiety Disorder-7; HAM-A, Hamilton Anxiety Rating Scale; HAM-D, Hamilton Depression Rating Scale; EPD-6, Brief Scale for the Assessment of Depressive Symptoms; EAL-20, Brief Scale for the Assessment of Anxiety Symptoms; BAI-I, Beck Anxiety Inventory; BDI, Beck Depression Inventory.
