# Supplementary material for: Pooled prevalence of anxiety and depressive symptoms among adults with type 2 diabetes: a systematic review and meta-analysis
Source: J Diabetes Metab Disord. 2026 Sep 24;25(2):265. doi: 10.1007/s40200-026-02079-z (PMC13612785; doi:10.1007/s40200-026-02079-z)
Supplement: Supplementary file 3 [file 40200_2026_2079_MOESM3_ESM.docx]

**Supplementary Material 3 Random-effects meta-analysis of the pooled prevalence of anxiety symptoms**

**Meta-analysis: proportion**

| Variable for studies | Study |
| --- | --- |
| Variable for total number of cases | Sample |
| Variable for the number of positive cases | Anxiety Anxiety |

| Study | Sample size | Proportion (%) | 95% CI | Weight (%) | |
| --- | --- | --- | --- | --- | --- |
|  |  |  |  | Fixed | Random |
| Albai et al. 2024 | 209 | 51.68 | 44.68 to 58.62 | 1.00 | 2.92 |
| Aldebani et al. 2024 | 412 | 47.09 | 42.18 to 52.04 | 1.96 | 2.96 |
| Aljohani et al. 2022 | 602 | 34.39 | 30.59 to 38.33 | 2.86 | 2.97 |
| ALkhayyal et al. 2025 | 391 | 84.91 | 80.97 to 88.31 | 1.86 | 2.95 |
| Al-Mohaimeed, 2017 | 300 | 43.67 | 37.98 to 49.49 | 1.43 | 2.94 |
| Alzahrani et al. 2019 | 450 | 38.00 | 33.50 to 42.66 | 2.14 | 2.96 |
| Baxi et al. 2025 | 200 | 30.00 | 23.74 to 36.87 | 0.95 | 2.91 |
| Butt et al. 2025 | 216 | 20.37 | 15.21 to 26.37 | 1.03 | 2.92 |
| Cardenas et al. 2022 | 208 | 33.65 | 27.27 to 40.51 | 0.99 | 2.92 |
| Dehesh et al. 2020 | 1500 | 62.00 | 59.49 to 64.46 | 7.13 | 2.99 |
| Elnaem et al.2025 | 606 | 41.58 | 37.63 to 45.62 | 2.88 | 2.97 |
| Ganasegeran et al. 2014 | 169 | 31.36 | 24.45 to 38.94 | 0.81 | 2.90 |
| Gashi et al. 2023 | 596 | 82.05 | 78.73 to 85.05 | 2.84 | 2.97 |
| Kaur et al. 2013 | 2508 | 30.62 | 28.82 to 32.47 | 11.91 | 2.99 |
| Khan et al. 2019 | 142 | 50.70 | 42.19 to 59.19 | 0.68 | 2.88 |
| Khuwaja et al. 2010 | 889 | 57.82 | 54.49 to 61.09 | 4.23 | 2.98 |
| Kintzoglanakis et al. 2022 | 182 | 17.58 | 12.35 to 23.91 | 0.87 | 2.91 |
| Masmoudi et al. 2013 | 62 | 48.39 | 35.50 to 61.44 | 0.30 | 2.75 |
| Mikaliukstiene et al. 2014 | 1022 | 42.37 | 39.32 to 45.47 | 4.86 | 2.98 |
| Mohana et al. 2023 | 100 | 30.00 | 21.24 to 39.98 | 0.48 | 2.84 |
| Nigussie et al. 2023 | 416 | 40.39 | 35.63 to 45.28 | 1.98 | 2.96 |
| Pardhan et al. 2024 | 600 | 22.00 | 18.75 to 25.53 | 2.85 | 2.97 |
| Paudel et al. 2023 | 283 | 31.45 | 26.08 to 37.21 | 1.35 | 2.94 |
| Palizgir et al. 2013 | 184 | 69.57 | 62.37 to 76.12 | 0.88 | 2.91 |
| Riise et al. 2025 | 1954 | 16.38 | 14.76 to 18.09 | 9.28 | 2.99 |
| Rosas-Matias et al. 2019 | 327 | 32.72 | 27.66 to 38.10 | 1.56 | 2.95 |
| Salih et al. 2022 | 380 | 79.21 | 74.78 to 83.18 | 1.81 | 2.95 |
| Sayin et al. 2019 | 636 | 48.43 | 44.48 to 52.39 | 3.02 | 2.97 |
| Sharma et al. 2021 | 296 | 49.66 | 43.83 to 55.51 | 1.41 | 2.94 |
| Subramanian et al. 2024 | 450 | 62.44 | 57.79 to 66.94 | 2.14 | 2.96 |
| Sun et al. 2016 | 893 | 56.10 | 52.78 to 59.39 | 4.25 | 2.98 |
| Tamene et al. 2026 | 409 | 36.19 | 31.52 to 41.05 | 1.95 | 2.96 |
| Tovila- Zárate et al. 2012 | 702 | 55.27 | 51.51 to 58.99 | 3.34 | 2.97 |
| Tripathi et al. 2026 | 2730 | 17.29 | 15.89 to 18.76 | 12.97 | 2.99 |
| Total (fixed effects) | 21024 | 39.42 | 38.75 to 40.08 | 100.00 | 100.00 |
| Total (random effects) | 21024 | 43.82 | 36.86 to 50.90 | 100.00 | 100.00 |

**Test for heterogeneity**

| Q | 3495.5632 |
| --- | --- |
| DF | 33 |
| Significance level | P < 0.0001 |
| I^2^ (inconsistency) | 99.06% |
| 95% CI for I^2^ | 98.93 to 99.16 |

**Publication bias**

| Egger's test | |
| --- | --- |
| Intercept | 7.9967 |
| 95% CI | -0.17 to 16.16 |
| Significance level | P = 0.0546 |
| Begg's test | |
| Kendall's Tau | -0,07493 |
| Significance level | P = 0.5332 |

**Supplementary Fig. 1 Funnel plot for publication bias assessment — anxiety symptoms**

Each circle represents an individual study. The x-axis shows the proportion (prevalence); the y-axis shows the standard error. Asymmetry in the plot may indicate publication bias, small-study effects, or genuine clinical/methodological heterogeneity.
