# Supplementary material for: Pooled prevalence of anxiety and depressive symptoms among adults with type 2 diabetes: a systematic review and meta-analysis
Source: J Diabetes Metab Disord. 2026 Sep 24;25(2):265. doi: 10.1007/s40200-026-02079-z (PMC13612785; doi:10.1007/s40200-026-02079-z)
Supplement: Supplementary file 4 [file 40200_2026_2079_MOESM4_ESM.docx]

**Supplementary Material 4 Random-effects meta-analysis of the pooled prevalence of depressive**

**Meta-analysis: proportion**

| Variable for studies | Study |
| --- | --- |
| Variable for total number of cases | Sample |
| Variable for the number of positive cases | Depression |

| Study | Sample size | Proportion (%) | 95% CI | Weight (%) | |
| --- | --- | --- | --- | --- | --- |
|  |  |  |  | Fixed | Random |
| Albai et al. 2024 | 209 | 57.42 | 50.41 to 64.21 | 1.00 | 2.92 |
| Aldebani et al. 2024 | 412 | 54.37 | 49.42 to 59.25 | 1.96 | 2.95 |
| Aljohani et al. 2022 | 602 | 3.32 | 2.04 to 5.08 | 2.86 | 2.96 |
| ALkhayyal et al. 2025 | 391 | 88.49 | 84.90 to 91.48 | 1.86 | 2.95 |
| Al-Mohaimeed, 2017 | 300 | 34.67 | 29.29 to 40.35 | 1.43 | 2.94 |
| Alzahrani et al. 2019 | 450 | 33.78 | 29.42 to 38.35 | 2.14 | 2.95 |
| Baxi et al. 2025 | 200 | 31.00 | 24.67 to 37.91 | 0.95 | 2.92 |
| Butt et al. 2025 | 216 | 25.00 | 19.37 to 31.32 | 1.03 | 2.93 |
| Cardenas et al. 2022 | 208 | 31.73 | 25.47 to 38.52 | 0.99 | 2.92 |
| Dehesh et al. 2020 | 1500 | 59.00 | 56.46 to 61.50 | 7.13 | 2.97 |
| Elnaem et al.2025 | 606 | 56.60 | 52.55 to 60.59 | 2.88 | 2.96 |
| Ganasegeran et al. 2014 | 169 | 40.24 | 32.78 to 48.04 | 0.81 | 2.91 |
| Gashi et al. 2023 | 596 | 73.99 | 70.27 to 77.47 | 2.84 | 2.96 |
| Kaur et al. 2013 | 2508 | 14.99 | 13.62 to 16.45 | 11.91 | 2.98 |
| Khan et al. 2019 | 142 | 49.30 | 40.81 to 57.81 | 0.68 | 2.90 |
| Khuwaja et al. 2010 | 889 | 43.53 | 40.24 to 46.86 | 4.23 | 2.97 |
| Kintzoglanakis et al. 2022 | 182 | 16.48 | 11.41 to 22.69 | 0.87 | 2.92 |
| Masmoudi et al. 2013 | 62 | 22.58 | 12.93 to 34.97 | 0.30 | 2.80 |
| Mikaliukstiene et al. 2014 | 1022 | 28.47 | 25.72 to 31.35 | 4.86 | 2.97 |
| Mohana et al. 2023 | 100 | 23.00 | 15.17 to 32.47 | 0.48 | 2.86 |
| Nigussie et al. 2023 | 416 | 42.31 | 37.51 to 47.22 | 1.98 | 2.95 |
| Pardhan et al. 2024 | 600 | 31.00 | 27.32 to 34.87 | 2.85 | 2.96 |
| Paudel et al. 2023 | 283 | 36.40 | 30.78 to 42.30 | 1.35 | 2.94 |
| Palizgir et al. 2013 | 184 | 70.65 | 63.50 to 77.12 | 0.88 | 2.92 |
| Riise et al. 2025 | 1954 | 12.18 | 10.76 to 13.71 | 9.28 | 2.98 |
| Rosas-Matias et al. 2019 | 327 | 23.85 | 19.34 to 28.85 | 1.56 | 2.94 |
| Salih et al. 2022 | 380 | 79.47 | 75.06 to 83.42 | 1.81 | 2.95 |
| Sayin et al. 2019 | 636 | 67.30 | 63.50 to 70.93 | 3.02 | 2.96 |
| Sharma et al. 2021 | 296 | 57.77 | 51.92 to 63.46 | 1.41 | 2.94 |
| Subramanian et al. 2024 | 450 | 72.89 | 68.53 to 76.94 | 2.14 | 2.95 |
| Sun et al. 2016 | 893 | 43.56 | 40.28 to 46.89 | 4.25 | 2.97 |
| Tamene et al. 2026 | 409 | 50.86 | 45.90 to 55.80 | 1.95 | 2.95 |
| Tovila- Zárate et al. 2012 | 702 | 47.72 | 43.97 to 51.49 | 3.34 | 2.96 |
| Tripathi et al. 2026 | 2730 | 20.29 | 18.80 to 21.85 | 12.97 | 2.98 |
| Total (fixed effects) | 21024 | 35.36 | 34.71 to 36.01 | 100.00 | 100.00 |
| Total (random effects) | 21024 | 41.83 | 33.83 to 50.06 | 100.00 | 100.00 |

**Test for heterogeneity**

| Q | 4753.4045 |
| --- | --- |
| DF | 33 |
| Significance level | P < 0.0001 |
| I^2^ (inconsistency) | 99.31% |
| 95% CI for I^2^ | 99.23 to 99.38 |

**Publication bias**

| Egger's test | |
| --- | --- |
| Intercept | 11.2739 |
| 95% CI | 2.03 to 20.52 |
| Significance level | P = 0.0184 |
| Begg's test | |
| Kendall's Tau | -0.01070 |
| Significance level | P = 0.9291 |

**Supplementary Fig. 2 Funnel plot for publication bias assessment — depressive symptoms**

Each circle represents an individual study. The x-axis shows the proportion (prevalence); the y-axis shows the standard error. Asymmetry in the plot may indicate publication bias, small-study effects, or genuine clinical/methodological heterogeneity.
